# Disseminated *Rothia kristinae* Infection: A Case Highlighting an Emerging Pathogen

**DOI:** 10.1155/carm/4049568

**Published:** 2026-01-29

**Authors:** Kody Dormire, Sravani Kamatam, Moni Roy, Sharjeel Ahmad

**Affiliations:** ^1^ Department of Internal Medicine, University of Illinois College of Medicine, Peoria, Illinois, USA, uic.edu; ^2^ Adult Hospitalist Services, OSF St Francis Medical Center, Peoria, Illinois, USA; ^3^ Section of Infectious Diseases, Department of Internal Medicine, University of Illinois College of Medicine, Peoria, Illinois, USA, uic.edu

**Keywords:** bacteremia, case report, discitis, endocarditis, *Rothia kristinae*

## Abstract

*Rothia kristinae* is usually found in the environment, on normal skin and mucosal surfaces of humans, and there is a limited medical literature available on this organism and its pathogenicity. Our case describes a woman in her early 70’s with left hip osteoarthritis presented with generalized weakness, fever, and altered mentation associated with left hip and back pain. Laboratory workup was significant for leukocytosis and elevated inflammatory markers. Blood culture demonstrated Gram‐positive cocci, later identified as *Rothia kristinae*. She was subsequently diagnosed with native aortic valve endocarditis, multilevel discitis, and left hip septic arthritis. She completed 2 weeks of intravenous vancomycin. Treatment was complicated by a drug reaction to vancomycin and switched to linezolid. She developed a left hip abscess and underwent left hip arthroplasty. Follow‐up transthoracic echocardiogram showed resolution of infective endocarditis. This case describes the extent of disseminated infection the organism can cause and its pathogenic potential warranting a low threshold for clinicians to initiate treatment.

## 1. Introduction

*Rothia kristinae* is a Gram‐positive, catalase‐positive, coagulase‐negative coccus found in the environment, on normal skin and mucosal surfaces of humans [1]. Formerly known as *Micrococcus kristinae*, in the genus Micrococcaceae, but was separated into the genera, which included the new genus *Kocuria* and later became known as *Kocuria kristinae*, with subsequent reclassification of the species into genus *Rothia* as *Rothia kristinae* [2]. It was first identified in the 1970s. With a paucity of available medical literature regarding this organism, our case report serves to further illustrate its pathogenic potential.

## 2. Case Presentation

A woman in her early 70’s with a history of diabetes and obesity presented at a local hospital with generalized weakness, altered mental status, low back pain, and left hip pain. The left hip pain started 2 weeks prior, 48 h after an intra‐articular corticosteroid injection. It was associated with fever and chills. This was associated with left‐sided flank pain, worse with movement, radiating to bilateral lower extremities. She had received treatment with levofloxacin for presumed *Escherichia coli* urinary tract infection without resolution of symptoms prior to admission to our hospital. Magnetic resonance imaging (MRI) of the lumbar spine showed discitis/osteomyelitis at L1‐L2 and L3‐L4, small peripherally enhancing psoas abscess bilaterally. Patient did have two blood cultures with growth of *Rothia kristinae* with repeat cultures showing no growth. Unfortunately, it was initially dismissed as a contaminant. Transthoracic echocardiogram (TTE) did not show any valvular vegetations. She was transferred to a tertiary care facility for further evaluation and management. Physical exam revealed limited movement of extremities due to diffuse myalgias and musculoskeletal pain consisting of shoulder, back pain, and hip pain. She had left hip greater trochanter tenderness on palpation and had limited mobility. Spinal exam showed midline tenderness over upper thoracic and lumbar spine.

**Figure 2:**
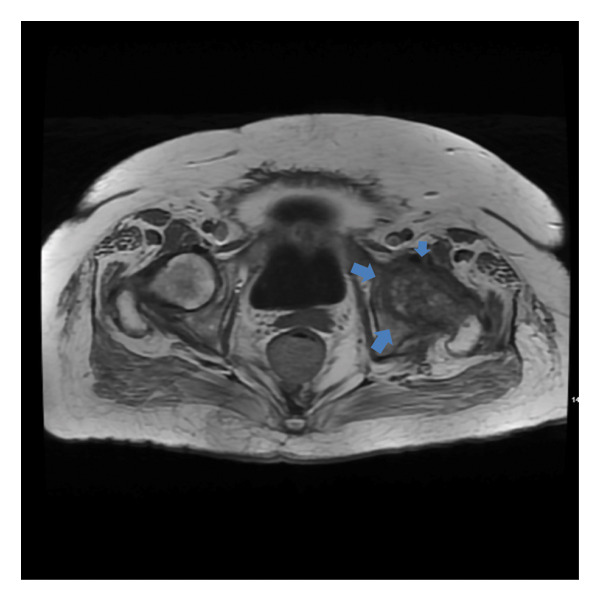
T1 Axial view on MRI of left hip with and without contrast showing patchy decreased T1 marrow signal (blue arrows) with increased T2 signal and enhancement centered within the left hip. Findings consistent with left hip septic arthritis and subjacent osteomyelitis.

Significant laboratory studies included a complete blood count showing a white blood count of 8.43 10^3^/mcL, C‐reactive protein of 10.86 mg/dL, and erythrocyte sedimentation rate of 45 mm/h. Hemoglobin A1c was 7. Second set of blood cultures speciated *Rothia kristinae* with penicillin resistance (MIC = 1 mcg/mL) and vancomycin susceptibility (MIC ≤ 0.5). She underwent a transesophageal echocardiogram, performed 13 days after the TTE revealing 1.2 × 3 × 0.28 cm vegetation on the aortic valve. A Positron Emission Tomography (PET) scan demonstrated numerous sites of uptake on T2‐T3, T6‐T7, T8‐T9, L1‐L2, and L3‐L4, consistent with discitis and osteomyelitis (Figure 1(a)). It also showed right glenohumeral, left sternoclavicular, and left hip septic arthritis, as shown in Figure 1(b). Nonspecific fludeoxygluocse (FDG) uptake in the right atrial appendage was also observed, with activity more over the noncoronary‐aortic‐valve cusp than the right coronary cusp raising concern for endocarditis (Figure 1(c)). Cardiothoracic surgery was consulted and recommended medical management with outpatient follow‐up after repeat echocardiography. Given PET findings, she had a repeat MRI of lumbar spine showing T2‐T3, T6‐T7, and T8‐T9 discitis and osteomyelitis, with dorsal epidural T8–T10 phlegmon and left hip multiloculated fluid collection within the left hip adductors along with findings suspicious for septic arthritis (Figure 2). An attempted left hip joint aspiration for synovial fluid analysis and culture sampling was unsuccessful.

Figure 1PET CT images with findings depicted in blue arrows: (a) Multifocal disc and adjoining endplate moderate tracer uptake at T2‐3, T6‐7, T 8‐9, L1‐2, L3‐4 spinal levels (blue arrows), suspicious for multifocal thoracic and lumbar spine discitis/osteomyelitis. (b) Moderately intense tracer activity at the right glenohumeral, left sternoclavicular, and left hip joints (blue arrows) is concerning for septic arthritis. (c) Nonspecific FDG uptake at the right atrial appendage (blue arrow) despite carbohydrate fasting raises concern for endocarditis.

**Figure:**
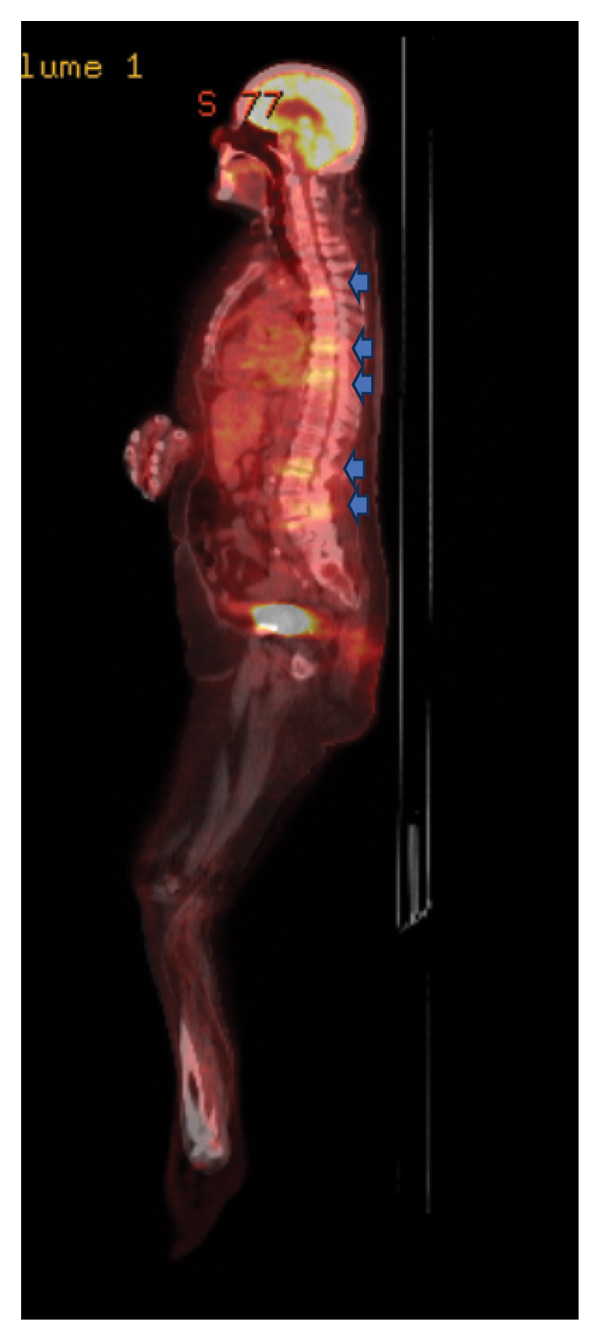
(a)

**Figure:**
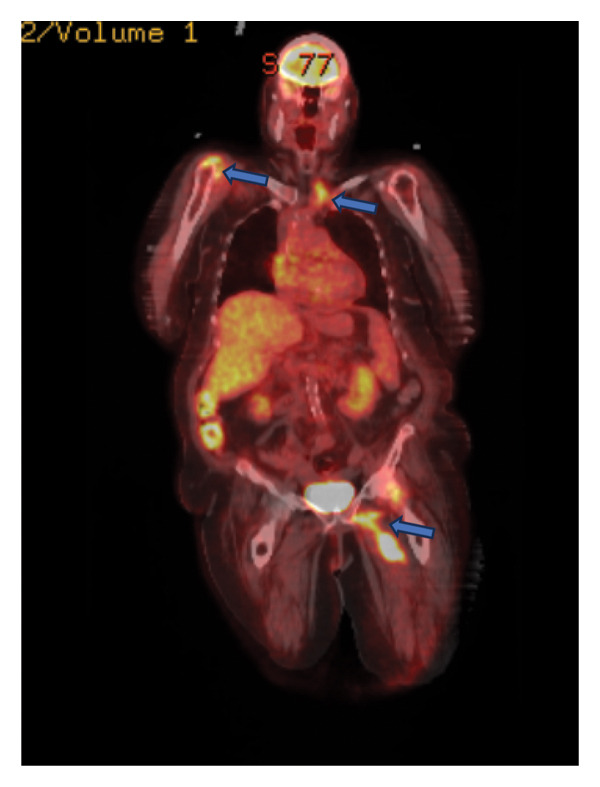
(b)

**Figure:**
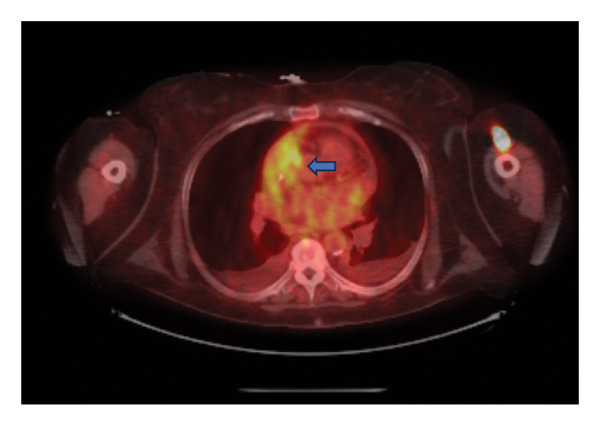
(c)

She was started on intravenous vancomycin and piperacillin‐tazobactam initially which was narrowed down to vancomycin monotherapy, once further microbiology data were available. She was discharged to a skilled nursing facility with plan for 6 weeks of treatment with this antibiotic, but she was readmitted 2 weeks later with acute kidney injury from acute tubular necrosis (ATN), peripheral eosinophilia, and diffuse drug rash secondary to vancomycin. She was transitioned to oral linezolid.

During readmission, a repeat MRI of left hip showed progressive septic arthritis and osteomyelitis with an adjacent abscess and patient proceeded with total left hip arthroplasty with the placement of a daptomycin spacer. Intraoperative cultures showed growth of *Rothia kristinae*. She was discharged on linezolid and completed the remaining 4 weeks of planned treatment. A follow‐up echocardiogram revealed no vegetation without aortic insufficiency. She returned to a skilled nursing facility and continued with physical rehabilitation.

## 3. Discussion

*Rothia kristinae* was initially named *Micrococcus kristinae* in 1974 by Kloss (after the person from which it was first isolated, Kristin Holding), and later, the name was changed to *Kocuria kristinae* and eventually to *Rothia kristinae* [3]. *Rothia* species belong to the family of Micrococcaceae, order Actinomyces, and class Actinobacteria. *Rothia kristinae* is a Gram‐positive, catalase‐positive, coagulase‐negative coccus and is a commensal of the skin and mucosal flora [4]. It is known to cause opportunistic infections in immunocompromised patients, including catheter‐associated infections and bacteremia [5]. Infective endocarditis in immunocompromised patients receiving total parenteral nutrition (TPN) has also been reported [6]. There are reported cases of endocarditis, synovial infections, and bacteremia due to *R. kristinae* in pregnant females, patients with acute leukemia, ovarian cancer, and on peritoneal dialysis [7–12].

The prevalence of *Rothia* infection is probably underestimated. This organism can be misidentified as Coagulase‐negative staphylococcus (CoNS), as they share phenotypical similarity or are disregarded as contaminants. The best way to identify CoNS is by genomic methods, such as RNA sequencing, which can distinguish it from *Rothia*. But given the labor‐intensive process and high costs, RNA sequencing may not be done in routine clinical practice. Vitek 2 system is fluorescence‐based rapid microbe analyzer that can provide quick results by identifying *Rothia* species and minimizing errors [2].

Due to the rarity of reported infections caused by *Rothia*, no standard of care regarding treatment and management exists. A small, predominant case report–based meta‐analysis by Živković Zarić et al. evaluated 28 patients with reported susceptibilities. All but one isolated specimen was susceptible to vancomycin, with susceptibilities to rifampicin, tigecycline, cefotaxime, and ampicillin/sulbactam [6]. All isolates were susceptible to linezolid, with noted penicillin, gentamicin, and erythromycin resistance. That being said that a few case reports documented the presence of multidrug‐resistant strains of *R. kristinae*. Isolate obtained from urine culture reported in case by Tewari et al. was found to be resistant to penicillin, erythromycin, trimethroprin‐sulfamethoxazole, ceftazidime, ceftriaxone, gentamicin, amikacin, oxacillin, ciprofloxacin, meropenem, imipenem, amoxicillin‐clavulanate, and vancomycin [13]. In a case report by Sivaraman et al., isolate was resistant to levofloxacin [14]. In a case reported by Laksmikanth et al., Rothia was noted to be sensitive to levofloxacin, minocycline, erythromycin, amikacin, and vancomycin. [15]. Ali et al. did report a case of infective endocarditis due to vancomycin‐resistant *R. kristinae* in a patient with a ventricular septal defect [16].

Our patient had bacteremia with metastatic foci of infection manifesting as endocarditis, septic arthritis, spinal osteomyelitis, and discitis, likely precipitated by left hip corticosteroid injection in this diabetic female. To our knowledge, this is the first reported case of R. *kristinae* septic arthritis after steroid injection. She was initially treated with vancomycin until a systemic drug reaction occurred, resulting in the need to transition to linezolid, which cleared the infection.

This case illustrates the pathogenic potential of this organism, resulting in invasive and severe disease. It also highlights the need for maintaining a high index of suspicion in the appropriate clinical scenario when this organism is isolated from a sample and initially dismissed as a contaminant.

## 4. Conclusion

*Rothia kristinae* is an emerging pathogen increasingly identified in bloodstream infections and endocarditis. As diagnostic methods improve, recognition of this organism will likely rise, making clinical vigilance and careful assessment essential for effective management.

## Author Contributions

Kody Dormire: responsible for patient management, collected clinical data, conducted the literature review, and wrote the initial draft of the manuscript.

Sravani Kamatam: collected patient data, performed literature review, contributed to manuscript writing and revisions, and figure preparation.

Moni Roy: provided patient care, performed critical revisions of the manuscript, and supervised the overall project.

Sharjeel Ahmad: provided patient care and performed critical revisions for important intellectual content.

## Funding

The authors received no specific funding for this work.

## Consent

Informed consent was obtained from the patient.

## Conflicts of Interest

The authors declare no conflicts of interest.

## Patient Perspective

The patient was offered the opportunity to contribute a perspective but declined.

## Data Availability

Data sharing is not applicable to this article as no datasets were generated or analyzed during the current study.
